# Two Different Inoculation Methods Unveiled the Relative Independence of DON Accumulation in Wheat Kernels from Disease Severity on Spike after Infection by Fusarium Head Blight

**DOI:** 10.3390/toxins13050353

**Published:** 2021-05-14

**Authors:** Rong Wang, Chen Hua, Yi Hu, Lei Li, Zhengxi Sun, Tao Li

**Affiliations:** Jiangsu Key Laboratory of Crop Genomics and Molecular Breeding/Key Laboratory of Plant Functional Genomics of the Ministry of Education/Collaborative-Innovation of Modern Crops and Food Crops in Jiangsu/Jiangsu Key Laboratory of Crop Genetics and Physiology, College of Agriculture, Yangzhou University, Yangzhou 225009, China; rongwang0602@foxmail.com (R.W.); chenhua0729@foxmail.com (C.H.); yihu0221@foxmail.com (Y.H.); lilei@yzu.edu.cn (L.L.); zhengxisun@yzu.edu.cn (Z.S.)

**Keywords:** wheat, fusarium head blight, disease severity, deoxynivalenol, overall resistance

## Abstract

Fusarium head blight (FHB) causes wheat yield loss and mycotoxin (deoxynivalenol, DON) accumulation in wheat kernel. Developing wheat cultivars with overall resistance to both FHB spread within a spike and DON accumulation in kernels is crucial for ensuring food security and food safety. Here, two relatively novel inoculation methods, bilateral floret inoculation (BFI) and basal rachis internode injection (BRII), were simultaneously employed to evaluate disease severity and DON content in kernels in a segregating population of recombinant inbred lines (RILs) developed from Ning 7840 (carrying *Fhb1*) and Clark (without *Fhb1*). Under both inoculation methods, four contrasting combinations of disease severity and DON content were identified: high severity/high DON (HSHD), high severity/low DON (HSLD), low severity/high DON (LSHD) and low severity/low DON (LSLD). Unexpectedly, the BRII method clearly indicated that disease severity was not necessarily relevant to DON concentration. The effects of *Fhb1* on disease severity, and on DON concentrations, agreed very well across the two methods. Several lines carrying *Fhb1* showed extremely higher severity and (or) DON content under both inoculation methods. The “Mahalanobis distance” (MD) method was used to rate overall resistance of a line by inclusion of both disease severity and DON content over both methods to select LSLD lines.

## 1. Introduction

Fusarium head blight (FHB) is one of the major wheat diseases caused by *Fusarium graminearum* (*Gibberella zeae* (Schw.) Petch) in the world [1]. The disease harms wheat root, stem and especially wheat spikes, and is frequently epidemic in China, Japan, Southeast Asia, the United States, Europe and Canada [2]. FHB causes accumulation of mycotoxin deoxynivalenol (DON) (also known as vomitoxin) and derivatives in kernels, which threatens human and animal health, thereby threatening food safety. Development of FHB-resistant cultivars to FHB is a priority approach to management of the devastating disease.

Five types of wheat FHB resistance have been proposed [3], including resistance to initial pathogen infection (type I), to pathogen spread within a spike (type II), to mycotoxin accumulation (type III), and to kernel infection (type IV), and tolerance to yield loss (type V). Type II and type III are the two most important types. DON is a sesquiterpene compound and is able to inhibit protein synthesis through interfering with ribosomal peptidyl transferase activity and hindering ribosomal circulation [4,5,6], and triggers a series of pathological reactions in humans and animals, such as appetite disorders, indigestion and immunosuppression [7,8,9]. DON is the most common mycotoxin in kernels in China, but also a frequent contaminant of wheat and other cereals around the world [10]. In 2008, a DON content of 18,000 μg·kg^−1^ was detected in kernels from a farm in Kansas, USA, causing a loss of 7.1 million bushels, worth $57 million [11]. DON was present in 66–70% of wheat kernels in northern and central Europe, and the maximum content was 8020 μg·kg^−1^ [12]. In 2010, 70% of 59 samples from Jiangsu and Anhui provinces in China were higher than 1000 μg·kg^−1^ in DON concentration, while 44% exceeded the maximum tolerable DON limits of the European Commission (1250 μg·kg^−1^) [13]. In Europe, the dominant chemotypes of mycotoxin were type B trichothecenes, including DON and its acetylated forms (3A-DON and 15A-DON), NIV and so on [14].

A glycosylated form of DON, DON-3-*O*-glucoside (D3G), is one of the masked mycotoxins. Previous studies have shown that D3G can be hydrolyzed to release toxin prototypes in the process of metabolism [15,16,17], so D3G is also harmful to human health and has been monitored as a routine contaminant in Europe, and the daily allowable intake (ADI) of D3G is the same as that of the prototype [18]. Development of FHB-resistant and low-toxin cultivars has become one of the key tasks in wheat breeding.

Due to the importance of FHB, intensive efforts have been put into breeding activities to decrease the disease severity. DON contents in kernels after harvest and food products have been monitored. However, few efforts were involved in lowering DON during practical breeding activities, even in those independent studies with *Fhb1* being involved. *Fhb1* has been recognized to be the most stable and effective QTL against both pathogens spread within a spike in different genetic background and DON accumulation in kernels [19,20]. A significant correlation between DON accumulation and FHB susceptibility was reported [21,22]; however, there were also reports of less DON in susceptible varieties [21,23]. The relationship between DON accumulation and disease severity remains unclear, probably due to different inoculation methods and genetic backgrounds.

Phenotyping method is critical to characterizing FHB resistance and to understanding the relationship between disease severity and DON accumulation during pathogen-host interactions. Currently, the methods frequently used for evaluation of FHB resistance include single-floret (spikelet) inoculation (SFI) [19], the misting-spray inoculation method [24], and spawn inoculation with pre-infected corn or oats [23]. The latter two methods mimic the natural infection, and more than one spikelet on a single spike may get infected at the stage of initial infection. The SFI method has been widely used to measure type II resistance, and only one spikelet is artificially infected. Different inoculation methods may produce a distinct relationship between disease severity and DON accumulation in kernels due to the variation of the number of infected spikelets. Here, we used two relatively novel methods for FHB inoculation, the Bilateral Florets Inoculation (BFI) method and the Basal Rachis Internode Injection (BRII) method. Compared with SFI, BFI injects inoculum into the two lateral florets of the fifth spikelet positioned from the spike base, thus increasing the infection rate to secure the successful inoculation. BRII was performed by injecting inoculum into the basal internodes of a rachis rather than into a floret. The greatest advantage of the BRII method over other existing methods is that no external misting facility is needed, because the humidity environment favoring disease development is provided by the water within plant tissue per se. When inoculated with the BRII method, some genotypes with a high level of resistance to FHB were visually symptomless on the spikelets [25]. Therefore, we were curious as to whether the two different inoculation methods generated similar patterns of DON content in the kernels from the infected spikes, which was of great help to understand the relationship between DON and disease severity. 

In this work, both disease severity and DON content in the kernels were assayed in a segregating population of inbred recombinant lines (RILs) developed from the cross of Ning7840 and Clark, and these two cultivars showed contrasting responses to FHB challenges under both the BFI method and the BRII method. The main purposes of this work were to: compare the patterns of disease severity and DON accumulation in kernels under the two methods to understand the relationship between disease severity and DON accumulation in kernels; estimate the effects of a specific QTL (*Fhb1* as a case) on disease severity and DON content under each method; develop a comprehensive evaluation strategy for rating the overall resistance of a variety to both disease severity and DON content under both methods; select the lines with a combination of low disease severity and low DON content for breeding highly resistant cultivars; and identify germplasm with different combinations of proportion of symptomatic spikelets in a spike (PSS) and DON content in kernels, which would be useful for deciphering the mechanism underlying disease spread and DON accumulation.

## 2. Results

### 2.1. The BFI and BRII Methods Produced Differential Patterns of Disease Severity

Under the BFI method, the PSS of Ning7840 and Clark were 0.05 and 0.89, respectively. The PSS of the RILs ranged from 0.04 to 1, with an average of 0.60. Under the BRII method, no diseased spikelets were observed in Ning7840, but the FHB symptom of Clark firstly appeared on the 3rd spikelet from the spike bottom, and finally spread to the whole spike. The PSS of the RILs ranged from 0 to 1 with an average of 0.43. The PSS of several lines (such as W-53 and W-142) was up to 1, which was significantly higher than that of the susceptible parent Clark (0.89), showing a significant transgressive segregation. 

Similar to Ning7840, some lines with a high resistance to FHB were visually symptomless under the BRII method, but the diseased spikelets were observed under the BFI method. Mean disease severity of the RILs under the BRII method was lower than that under the BFI method, but there were some exceptions, such as W-173, which had significantly higher PSS under the BRII method (0.75) than that under BFI method (0.08).

**Figure 1:**
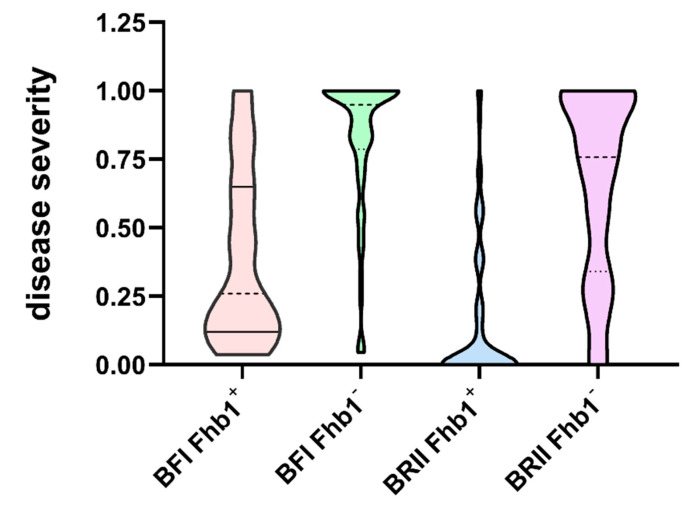
Proportion of symptomatic spikelet (PSS) of the RILs from the segregating population under the BFI and the BRII inoculation methods.

The differences in PSS and DON between the contrasting alleles of *Fhb1* under the two inoculation methods were compared to understand the consequences of inoculation methods on the genetic effects. Under the BFI method, the disease severities of the RILs with *Fhb1* ranged from 0.04 to 1.0, with an average PSS of 0.38, and those lines without *Fhb1* ranged from 0 to 1.0, with an average PSS of 0.81. Under the BRII method, the disease severities of the RILs with *Fhb1* ranged from 0 to 1.0, with an average PSS of 0.16, and those lines without *Fhb1* had the same range but with an average PSS of 0.66 (Figure 1). The mean PSS of the lines carrying *Fhb1* was significantly lower than that of the lines without *Fhb1* across the two methods (Table 1); however, the difference between the two contrasting alleles of *Fhb1* was almost identical, indicating that both methods could be used to estimate the genetic effect of QTL such as *Fhb1* (Table 1).

### 2.2. The BFI and BRII Methods Generated Similar Patterns of DON Content in Kernels

**Figure 2:**
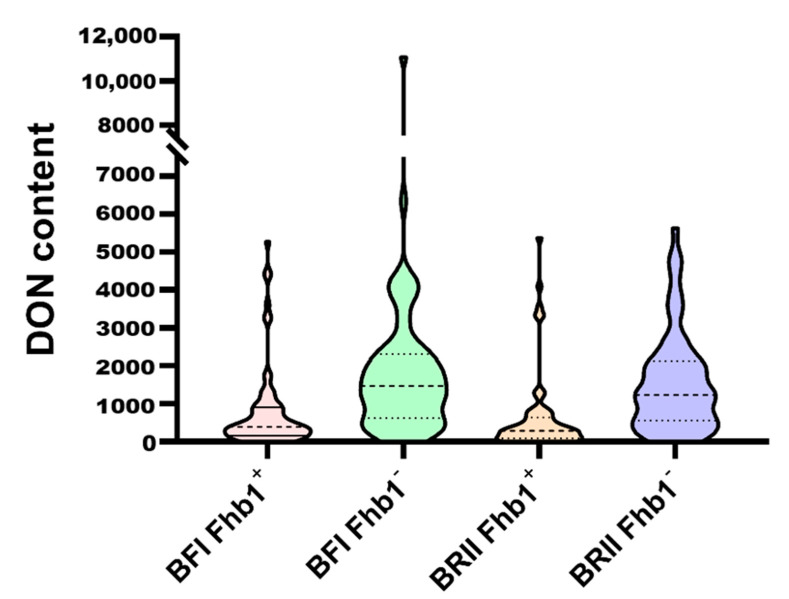
DON content of the RILs from the segregating population under the BFI and the BRII inoculation methods.

DON content in the kernels of Ning7840 and Clark was 589.4 μg·kg^−1^ and 956.0 μg·kg^−1^, respectively, under the BFI method, and the DON content in kernels of the RILs ranged from 0 μg·kg^−1^ to 11,081.8 μg·kg^−1^, with an average DON content of 1493.6 μg·kg^−1^. Under the BRII method, DON concentration in kernels significantly differed between Ning7840 (260.76 μg·kg^−1^) and Clark (1152.30 μg·kg^−1^). DON concentrations in kernels of the RILs ranged from 0 μg·kg^−1^ to 5630.50 μg·kg^−1^, with an average of 1098.38 μg·kg^−1^. DON concentrations under both inoculation methods showed a significant transgressive segregation. Despite a significant positive correlation in DON content between the two inoculation methods (Table 2), some lines, such as W-81 and W-149, had a large difference in DON content between the two methods, differing by 9710.2 μg·kg^−1^ and 5342.2 μg·kg^−1^, respectively. Some near-isogenic line pairs (NIL) derived from a heterozygote were significantly different across the two inoculation methods; for example, DON content of W-106 (899.8 μg·kg^−1^) was about nine times of that of W-105 (96.05 μg·kg^−1^), and DON content in W-12 (881.05 μg·kg^−1^) was twofold that of W-13 (312.3 μg·kg^−1^) (Table S1). Mean DON content of the lines carrying *Fhb1* was significantly lower than that of the lines without *Fhb1* between the two inoculation methods (Figure 2), and the difference in DON contents between the two contrasting alleles of *Fhb1* under the BFI method (987.67 μg·kg^−1^) was also close to that under the BRII method (916.59 μg·kg^−1^), suggesting the pleiotropic effects of *Fhb1* (Table 1).

The *F. graminearum* strain used in this experiment was 15A-DON chemotype, and 3A-DON was not detected in all lines. The detection rates of D3G and 15A-DON in the RILs were about 10% and 30%, respectively. The contents of D3G and 15A-DON were also very low across the two methods, and the maximum contents of D3G and 15A-DON were less than 100 μg·kg^−1^, and the mean values ranged from 0.81 to 1.76 ppb (Table S2). There was no significant correlation between D3G and 15A-DON contents between the two inoculation methods. The differences in D3G and 15A-DON contents were not significant between the two contrasting alleles of *Fhb1* across the two methods, showing that *Fhb1* might be not involved in glycosylation or acetylization of DON (Table S3).

### 2.3. Low Disease Severity Does Not Mean Low DON Content

Despite the significant correlation between PSS and DON (*p* < 0.01) across the BFI and BRII methods, some lines had low PSS (lower than the average) but high DON content in kernels (higher than the average) (Table S1) or vice versa. To better understand combinations of PSS and DON concentration, we classified those lines into four contrasting combinations of severity and DON: two typical combinations of high severity/high DON (HSHD) and low severity/low DON (LSLD), and two atypical combinations of high severity/low DON (HSLD) and low severity/high DON (LSHD). One of the atypical lines (such as W-66) had the highest PSS up to 1.0, but no DON was detected in kernels under both methods, and another atypical line (such as W-116) did not have visible diseased spikelets under the BRII method, but DON content in the kernels was up to 5373.17 μg·kg^−1^ (Table S1).

On average, PSS and DON contents of the lines with *Fhb1* were significantly lower than those of the lines without *Fhb1* (*p* < 0.01) (Table 1). Unexpectedly, nine lines carrying *Fhb1* showed highly susceptible to FHB (PSS > 0.75), and one of them also had high DON content up to 3000 μg·kg^−1^. Five lines without *Fhb1* showed high resistance to FHB (PSS < 0.25), and two of them had low DON content, lower than 200 μg·kg^−1^.

### 2.4. Evaluation of Overall Resistance and Selection of LSLD Lines

By inclusion of disease severity and DON content with equal weight over the two methods, a comprehensive evaluation of the overall resistance of each line was performed by calculating the Mahalanobis distance of each line to the postulated reference. The smaller the Mahalanobis distance to the reference, the higher the resistance to both diseases spread and to DON accumulation in kernels (Table S3). Among the RILs, 31 lines had closer distance to the postulated reference than did the resistant parent Ning7840, and four lines were almost similar to the reference, and 67 lines had longer distances to the reference than the susceptible parent Clark; the majority of these lines had higher DON concentrations than Clark, showing a transgressive segregation in overall resistance. Unexpectedly, 11 out of 67 lines with the resistant allele at *Fhb1* either had higher disease severity or higher DON concentration or both (Table S3), suggesting that *Fhb1* did not work in specific genetic backgrounds.

## 3. Discussion

### 3.1. Complex Relationship between Disease Severity and DON Concentration in Kernels

Many studies showed that there was a positive correlation between disease severity and DON content in kernels [26,27]. However, there have been also reports about low DON in high susceptible varieties, that is, no or weak correlation between DON content and PSS [28,29]. Both inoculation methods in the current study suggested that PSS and DON content in kernels were significantly positively correlated (*p* < 0.001), whereas two atypical combinations, HSLD and LSHD, were also discovered, and in particular, under the BRII method, some lines did not show any symptomatic trait on spikelets but DON were up to 5000 μg·kg^−1^ in the kernels. These facts suggested that disease infection and DON accumulation had a causal relationship, whereas disease severity and DON accumulations in kernels had relatively independent mechanisms in planta. In addition, the BRII inoculation method also indicated that DON was induced in rachis and then transported to kernels.

Previous studies [30,31] suggested that the node of rachilla plays a decisive role in shaping type II resistance, and that resistant varieties have a rapid response mechanism at the early stage of infection, which reduces DON accumulation by forming papillae, increasing host cell wall thickness, lignin or (and) other measures to resist mycelium invasion and spread within the host, so DON content of a resistant variety is expected to be lower than that of a susceptible variety. However, the results in this study clearly showed that some lines with *Fhb1* had visible diseased spikelets under the BRII method, indicating that the node was not decisive for FHB resistance mediated by *Fhb1*.

### 3.2. Different Inoculation Methods Did Not Alter the Effects of Fhb1 on PSS and DON

FHB is a typical quantitative trait controlled by multiple genes. *Fhb1* has the greatest effect on type II resistance to FHB among all QTL identified so far [32]. The single QTL of *Fhb1* explains 15% to 30% of phenotypic variation [33]. Therefore, we used the effects of *Fhb1* on disease severity and DON content to evaluate the practicability of the two inoculation methods. Under the two inoculation methods, the average disease severity and DON content of the lines carrying *Fhb1* were significantly lower than those of the lines without *Fhb1*. Interestingly, the difference in both PSS and DON concentration between the two contrasting alleles of *Fhb1* were almost identical, suggesting that the combination of two inoculation methods could be used for cross-validation and increase phenotypic accuracy, and consequently speed up the genetic mapping and positional cloning genes of interest.

Several previous studies claimed that *Fhb1* was the only QTL with stable type II resistance in different environments and genetic backgrounds [34,35]. Unexpectedly, both inoculation methods consistently demonstrated that some lines (Table S4) with *Fhb1* belonged to the HSHD category, clearly indicating that *Fhb1* was negatively regulated in some genetic backgrounds. The existence of inhibitor(s) of *Fhb1* may distract researchers away from targeting the real causal gene, which may partially, if not entirely, explain the conflicting results of three papers [36,37,38] on *Fhb1* cloning. Identification and elimination of inhibitor (s) of *Fhb1* would be also crucial for genetic improvement of FHB resistance and DON accumulation.

### 3.3. No Correlations among 15A-DON, D3G and DON

DON interferes with protein translation through binding to the α site at the center of the ribosomal 60s subunit peptidyl transferase in eukaryotic nuclei [39,40]. 15A-DON and DON-3- β-d-glucoside (D3G) are the derivatives of DON. Some studies indicated that the glycosylation of DON (UGT) reaction is an important detoxification mechanism in plants [22]; however, there have also been reports that D3G can be hydrolyzed to release toxin prototypes in the process of metabolism, so D3G is also harmful to human health and has been monitored as a routine contaminant in Europe, and the daily allowable intake (ADI) of D3G is the same as that of the prototype [17,18]. Previous reports showed that there were highly significant relationships among PSS, DON and D3G content in wheat [41,42]. However, our study showed that the detected rate of D3G was very low, and there was no correlation among D3G,15A-DON and DON contents across the two inoculation methods, and no significant differences were identified either between the contrasting alleles of *Fhb1*, suggesting that *Fhb1* may not be involved in glycosylation or acetylation of DON.

### 3.4. Overall Resistance to FHB Should Be More Useful and Reasonable in Wheat Breeding

In breeding practice, wheat FHB resistance has been evaluated according to the disease severity or disease index [43], and DON concentration has seldomly been considered in practical breeding objectives. One of the possible reasons was that FHB disease severity is relatively easily scored and DON assay is much more expensive, technically difficult and time-consuming. DON concentration in food and feed is usually monitored after harvest. Although FHB infection and DON accumulation are causally related, FHB severity and DON concentration were relatively independent, and low FHB severity did not mean low DON concentration. The lines with low FHB severity but high DON concentration are useless either in food or in feed. Therefore, comprehensive evaluation, by inclusion of both FHB severity and DON concentration, of overall resistance to FHB is strongly recommended during wheat breeding practice.

The Mahalanobis distance has been used in various models, and the Mahalanobis distance technique relies on multivariate mean and covariance matrix [44]. To evaluate overall resistance and to select LSLD lines, a postulated line with the best resistance was proposed as a reference. The Mahalanobis distance statistic (D^2^) was used to calculate the similarity (or distance) of each line to the reference by inclusion of both FHB severity and DON kernels over both inoculation methods. Interestingly, 31 lines had closer overall resistance to the reference than the resistant parent Ning7840 and could be used in breeding practice. Unexpectedly, 11 lines with the resistant *Fhb1* allele showed either high disease severity or high DON concentration or both across both inoculation methods, suggesting that negative regulator(s) of *Fhb1* was (were) most likely present in these lines, and removal of the potential inhibitors of *Fhb1* was necessary during the introduction of *Fhb1*. Unfortunately, up to now, no inhibitors of *Fhb1* have been reported. Interestingly, three lines without *Fhb1* showed typical FHB symptom under both the BFI and BRII methods, but no DON were detected in kernels, and these lines could be used for untangling the complex relationship between disease severity and DON accumulation.

## 4. Conclusions

The BRII inoculation method indicated that there was a causal relationship between *F. graminearum* infection and DON accumulation, whereas disease severity and DON were relatively independent. In addition, two atypical combinations, HSLD and LSHD, were identified across both inoculation methods, highlighting the necessity of overall resistance evaluation by inclusion of both disease severity and DON concentration in breeding practice. Furthermore, several lines with *Fhb1* showing HSHD phenotype across both methods suggested that identification and elimination of *Fhb1* inhibitors is critically important when using *Fhb1* in a wheat breeding program. Those lines with typical and atypical combinations of disease severity and DON concentration would be also valuable for genetically untangling the complex relationship of disease severity and DON content and breeding LSLD lines to benefit both food security and food safety.

## 5. Materials and Methods

### 5.1. Materials

A segregating population consisting of 145 recombinant inbred lines (RILs, F14) was developed from the cross of Ning7840 and Clark. The segregating population of Ning7840/Clark (F7) was kindly provided by Dr. Guihua Bai at Kansas State University. Ning7840 carries *Fhb1* and shows moderate-to-high resistance to FHB, and Clark does not carry any known resistant QTL and showed high susceptibility to FHB [45,46]. The two parents and the RILs were planted in the experimental field of Yangzhou University from October 2018 to June 2019, with 15 plants in each line and two replicates per line.

### 5.2. Fusarium graminearum Strain, Inoculum Preparation and FHB Inoculation

A highly pathogenic *Fusarium graminearum* strain F1312 (producing 15A-DON chemotype) was used for inoculation. The strain was collected in a wheat field in Jiangsu Province, and provided by Professor Huigu Chen from the Jiangsu Academy of Agricultural Sciences.

The strain was firstly activated on potato dextrose agar (PDA) medium. Five blocks (6 mm in diameter) of the activated strain were then added to a 200 mL of flask filled with 100 mL sterilized mung bean soup for induction of macrospore. After 3–5 days of incubation at 15× *g*, 25 °C on a shaker, the inoculum were diluted to a concentration of 1 ×10^5^ spores per microlitre (μL) for a Bilateral Florets Inoculation (BFI) method, and 1 ×10^6^ spores per microlitre for a Basal Rachis Internode Injection (BRII) method. The spores were sub-packed into a 2 mL centrifuge tubes and stored at 4 °C for use.

Both BFI and BRII methods were used for phenotyping. The BFI method was performed by injecting 10 μL of the inoculum into the bilateral florets of the fifth spikelet positioned from the bottom of a spike at flowering period of wheat. Ten to 15 spikes were inoculated for each line per replicate. The BRII method was performed by injecting about 1 μL of concentrated inoculum into the basal rachis internode of a spike, and 10 to 15 spikes were inoculated at anthesis of wheat for each line per replicate.

### 5.3. Phenotypic Evaluation

For the BFI method, the disease severity was estimated by the proportion of symptomatic spikelet in a spike (PSS, calculated by ∑ (number of diseased spikelet of per spike/total spikelet)/total number of spikelets) in 21 days of inoculation. For the BRII method, the phenotypic data was scored in a qualitative–quantitative way. The rachis internode got infected but no visible diseased spikelet was qualitatively scored as the resistant (R) and those with visible diseased spikes as the susceptible (S), and in the latter case, the disease severity was further scored quantitatively as under the BFI method.

### 5.4. Determination of DON Contents and Its Derivative Forms

All the inoculated spikes of the same line were harvested and threshed manually. The stripped kernels were ground into powder and 1g powder was weighed for determination of DON content. Triple Quadruopole LC/MS/MS (TSQ-Vantage, Thermo Fisher SCIENTIFIC, USA) was used to measure DON, 3A-DON, 15A-DON and D3G. All mycotoxin standards were purchased from Romer Labs (Tulln, Austria). Determination of mycotoxin contents in kernels followed the protocol by Mao et al. [47]. The parameters of mass spectrometry are detailed in Supplementary Material (Table S4: Technical Note).

### 5.5. Classification of Disease Severity and DON

A quartile method was used to classify FHB phenotypic data under the two inoculation methods. PSS under each method was classified into four categories, with each category accounting for 25% of the total (Table 3). For the disease severity, the first 25% was defined as high resistance to FHB (HR), the second quartile from 25% to 50% was defined as moderate resistance to FHB (MR), the third quartile from 50% to 75% was defined as moderate susceptibility to FHB (MS), and the fourth quartile greater than 75% was defined as high susceptibility to FHB (HS). DON concentration under each method was also classified in a similar way, with each category accounting for 25% of the total (Table 3). From the perspectives of safe consumption, compatibility across countries and practicability, we modified the definition of DON levels: the first 25% was defined as low DON (LD). However, a DON content less than 1000 μg·kg^−1^ in kernels was considered as an acceptable DON level since 1000 μg·kg^−1^ is a legal limit in unprocessed cereals in China (more lenient in unprocessed cereals in USA, Europe and Canada); a DON content from 1000 μg·kg^−1^ to the upper limit of the third quartile (75%) was considered as a risky DON level; and the fourth quartile greater than 75% was considered as an dangerous DON level (herein called high DON level, HD).

### 5.6. Evaluation of Overall Resistance to FHB

The Mahalanobis distance (MD) method [48] was used for evaluating the overall resistance to FHB for any line by equally weighted inclusion of the disease severities and DON contents over the two inoculation methods. A postulated line with zero disease severity and zero DON content over the two methods was used as a reference for the best resistance. The MD of each line to the postulated reference was calculated.

### 5.7. DNA Extraction and Fhb1 Genotyping

DNA was extracted from fresh wheat leaves of the two parents and 145 RILs using the modified CTAB method [49]. The DNA quality were determined by agarose gel electrophoresis. *Fhb1*-associated gene marker and amplication conditions followed the protocol by Su et al. [50].

### 5.8. Data Processing

Excel 2016 (Microsoft Office Inc., Redmond, WA, USA) was used for data processing. SPSS statistics 23 (SPSS Inc., Chicago, IL, USA) was used for variance analysis and correlation analysis. GraphPad Prism 8 (GraphPad Software Inc., San Diego, CA, USA) was used for making figures.

## Figures and Tables

**Table 1:** Differences in PSS and DON between the two contrasting alleles of *Fhb1* under the BFI and the BRII methods.

Trait	Methods	*Fhb1*	Min	Max	Mean Value	Allelic Difference	DF	SD	T Value	Sig.
PSS	BFI	Yes	0.04	1.00	0.38	0.43	70	0.40	−8.98	*p* < 0.01
		No	0	1.00	0.81					
	BRII	Yes	0	1.00	0.16	0.50	70	0.44	−9.47	*p* < 0.01
		No	0	1.00	0.66					
DON (μg·kg^−1^)	BFI	Yes	0	5281.60	787.60	987.67	70	1979.22	−4.21	*p* < 0.01
		No	0	11081.80	1775.26					
	BRII	Yes	0	5373.17	554.03	*916*.59	70	1465.02	−5.20	*p* < 0.01
		No	0	5630.47	1470.61					

**Table 2:** Correlations between PSS and DON contents under the BFI and the BRII methods.

	BRII-PSS	BFI-DON	BRII-DON
BFI-PSS	0.633 **	0.427 **	0.386 **
BRII-PSS		0.331 **	0.336 **
BFI-DON			0.455 **

** significantly correlated at 0.01 level.

**Table 3:** Quantile thresholds of PSS and DON under the BFI and the BRII methods.

	PSS		DON	
	BFI	BRII	BFI	BRII
Min	0.04	0.00	0.00	0.00
25%	0.21	0.00	326.96	212.84
50%	0.67	0.33	883.31	620.44
75%	1.00	0.84	1722.30	1378.63
Max	1.00	1.00	11081.75	5630.47

## Data Availability

The data presented in this study are available on request from the corresponding author.

## Supplementary Materials

The following are available online at https://www.mdpi.com/article/10.3390/toxins13050353/s1, Table S1: Representative lines with contrasting combinations of PSS and DON under the BFI and the BRII methods. Table S2: Differences in D3G and 15A-DON between the two contrasting alleles of Fhb1 under the BFI and the BRII methods. Table S3: Overall resistance based on the Mahalanobis distance to the postulated reference. Table S4: Technical Note: The parameters of mass spectrometry.
