# Supplementary material for: Two Different Inoculation Methods Unveiled the Relative Independence of DON Accumulation in Wheat Kernels from Disease Severity on Spike after Infection by Fusarium Head Blight
Source: Toxins (Basel). 2021 May 14;13(5):353. doi: 10.3390/toxins13050353 (PMC8156083; doi:10.3390/toxins13050353)
Supplement: Supplementary file 1 [file toxins-13-00353-s001.zip › toxins-1223582-SI.pdf]

Supplementary Materials: Two Different Inoculation Methods Unveiled the Relative Independence of DON Accumulation in Wheat Kernels from Disease Severity on Spike after Infection by Fusarium Head Blight

Rong Wang, Chen Hua, Yi Hu, Lei Li, Zhengxi Sun and Tao Li*

Table S1. Representative lines with contrasting combinations of PSS and DON under the BFI and the BRII methods.

Serial Number	*Fhb1* Stauts	PSS		DON		3A-DON		15A-DON		D3G		Characteristics
		BFI	BRII	BFI	BRII	BFI	BRII	BFI	BRII	BFI	BRII	
Ning7840	+	0.05	0.00	589.35	260.76	NF	NF	NF	11.69	2.82	NF	
Clark	-	0.89	0.59	955.96	1152.30	NF	NF	NF	12.26	NF	NF	
W-104	+	0.11	0.00	10.78	0.00	NF	NF	NF	13.07	NF	NF	LSLD
W-105	+	0.07	0.00	108.79	83.31	NF	NF	18.94	11.22	2.88	NF	LSLD
W-114	+	0.04	0.00	0.00	0.00	NF	NF	29.28	14.34	NF	NF	LSLD
W-141	+	0.04	0.00	125.06	21.27	NF	NF	NF	NF	NF	2.82	LSLD
W-145	+	0.04	0.00	253.31	80.89	NF	NF	16.42	16.47	NF	9.44	LSLD
W-164	+	0.06	0.00	0.00	0.00	NF	NF	17.21	NF	NF	NF	LSLD
W-165	+	0.20	0.00	0.00	0.00	NF	NF	26.50	14.35	NF	2.86	LSLD
W-24	+	0.05	0.00	111.69	151.49	NF	NF	11.67	11.13	3.24	3.86	LSLD
W-52	+	0.21	0.00	1226.44	1169.66	NF	NF	40.82	NF	NF	NF	LSHD
W-11	-	1.00	1.00	4247.57	2094.47	NF	NF	NF	24.99	NF	NF	HSHD
W-17	-	1.00	1.00	1768.83	2682.06	NF	NF	NF	NF	NF	NF	HSHD
W-64	+	1.00	1.00	181.36	197.78	NF	NF	14.62	12.56	2.94	NF	HSLD
W-53	+	1.00	1.00	70.08	408.59	NF	NF	11.01	12.84	NF	NF	HSLD
W-67	+	0.69	0.71	4375.42	210.69	NF	NF	11.10	12.91	NF	NF	BFI-DON>> BRII-DON
W-76	-	1.00	1.00	208.88	4787.28	NF	NF	11.89	NF	3.79	NF	BFI-DON<< BRII-DON

W-81	-	0.79	1.00	#######	1371.57	NF	NF	NF	NF	NF	NF	BFI-DON＞＞ BRII-DON
W-149	-	1.00	1.00	6341.28	999.03	NF	NF	NF	54.19	NF	NF	BFI-DON＞＞ BRII-DON

NF: not detected; +: with Fhb1,-: without Fhb1; HSHD: high severity/high DON; HSLD: high severity /low DON; LSHD: low severity /high DON; LSLD: low severity/low DON**.**

Table S2. Differences in D3G and 15A-DON between the two contrasting alleles of *Fhb1* under the BFI and the BRII methods.

Trait	Methods	*Fhb1*	Minimum	Maximum	Average	Difference of Average	Standard Deviation	T Value	df	Significance
D3G	BFI	Yes	0.00	10.90	4.52	1.05	2.82	1.29	13.66	0.22
		No	0.00	12.30	3.48		0.56			
	BRII	Yes	0.00	66.30	6.52	3.74	13.01	−0.68	29.00	0.50
		No	0.00	36.10	10.26		12.18			
15A-DON	BFI	Yes	0.00	100.60	24.95	8.83	21.48	−1.08	41.50	0.29
		No	0.00	165.90	33.78		41.69			
	BRII	Yes	0.00	88.60	14.21	7.16	11.64	−2.48	71.76	0.02
		No	0.00	65.80	21.37		15.08			

Table S3. Overall resistance based on Mahalanobis distance to the postulated reference.

Entry	*Fhb1* Genotype	BFI-PSS	BRII-PSS	BFI-DON	BRII-DON	Mahalanobis Distance (d^2)
Ref		0.00	0.00	0	0	0.000
W-109	+	0.56	0.00	0.0	0.0	0.000
W-114	+	0.04	0.00	0.0	0.0	0.000
W-164	+	0.06	0.00	0.0	0.0	0.000
W-165	+	0.20	0.00	0.0	0.0	0.000

W-104	+	0.11	0.00	10.8	0.0	0.000
W-105	+	0.07	0.00	108.8	83.3	0.008
W-141	+	0.04	0.00	125.1	21.3	0.008
W-126	+	0.43	0.00	109.1	94.2	0.009
W-32	+	0.26	0.00	107.6	107.6	0.011
W-24	+	0.05	0.00	111.7	151.5	0.019
W-145	+	0.04	0.00	253.3	80.9	0.033
W-120	+	0.07	0.00	177.7	216.5	0.039
W-161	+	0.30	0.00	236.3	236.3	0.052
W-26	-	0.45	0.00	309.4	222.6	0.062
W-21	+	0.13	0.00	118.1	287.3	0.064
W-168	+	0.46	0.07	338.6	103.8	0.065
W-154	+	0.10	0.00	341.0	211.6	0.068
W-42	+	0.16	0.00	352.0	25.5	0.070
W-151	+	0.17	0.00	292.9	281.8	0.075
W-27	+	0.12	0.00	319.1	274.0	0.078
W-146	+	0.14	0.00	265.3	301.9	0.078
W-113	+	0.17	0.00	344.4	332.6	0.105
W-51	+	0.10	0.00	317.3	349.2	0.107
W-13	+	0.22	0.00	259.3	365.3	0.107
W-143	+	0.18	0.00	424.0	294.6	0.113
W-112	+	0.20	0.00	358.3	358.3	0.119
W-133	+	0.67	0.00	523.9	207.7	0.140
W-150	+	0.07	0.00	392.4	413.4	0.153
W-115	+	0.18	0.00	395.6	439.8	0.168
W-3	+	0.59	0.00	446.8	437.9	0.180
W-1	+	0.05	0.00	589.3	260.8	0.180
W-47	+	0.30	0.02	453.7	517.6	0.213
W-46	+	0.84	0.13	648.7	325.6	0.231
W-122	+	0.81	0.12	524.2	523.1	0.238

W-135	+	0.63	0.20	437.6	216.7	0.274
W-117	-	0.25	0.19	427.4	515.3	0.317
W-130	+	0.58	0.00	587.2	600.7	0.329
W-155	+	0.22	0.00	608.8	598.3	0.335
W-170	+	0.19	0.22	0.0	0.0	0.371
W-55	+	0.10	0.22	679.5	250.9	0.406
W-139	+	0.05	0.00	884.8	63.7	0.445
W-175	+	0.04	0.04	745.8	759.9	0.479
W-144	+	0.45	0.00	362.9	820.4	0.523
W-119	+	0.85	0.00	755.0	750.6	0.523
W-147	+	0.16	0.00	1002.1	165.5	0.534
W-177	-	1.00	0.27	426.2	563.0	0.535
W-163	+	0.17	0.00	826.9	826.9	0.632
W-93	-	0.04	0.16	925.1	915.6	0.674
W-54	+	0.41	0.02	1097.7	49.7	0.684
W-12	+	0.46	0.00	923.0	839.1	0.698
W-16	+	0.81	0.19	1198.5	687.0	0.722
W-106	+	0.26	0.00	916.1	883.5	0.740
W-91	+	0.83	0.36	296.6	327.2	0.837
W-129	-	1.00	0.26	1060.2	38.0	0.893
W-66	-	0.82	0.35	0.0	0.0	0.941
W-73	+	0.82	0.37	63.9	0.0	0.982
W-102	-	0.41	0.09	1144.8	1116.8	1.025
W-166	+	0.22	0.06	242.3	1182.9	1.095
W-100	-	0.86	0.00	1144.9	1130.4	1.192
W-121	+	0.42	0.42	147.7	147.7	1.192
W-86	+	0.16	0.40	0.0	0.0	1.223
W-52	+	0.21	0.00	1226.4	1169.7	1.307
W-140	-	0.70	0.31	1259.6	1191.3	1.311
W-22	+	0.06	0.00	941.1	1334.6	1.424

W-103	-	1.00	0.16	1417.2	1417.2	1.588
W-43	+	1.00	0.40	1287.5	98.0	1.627
W-71	+	0.07	0.06	1776.5	188.2	1.663
W-29	-	1.00	0.38	527.4	1385.7	1.798
W-174	-	0.05	0.50	0.0	0.0	1.877
W-23	-	1.00	0.26	1984.6	550.7	1.886
W-152	+	0.45	0.00	1428.7	1428.7	1.888
W-87	-	1.00	0.55	626.2	686.1	1.963
W-25	+	0.24	0.55	447.9	649.3	1.968
W-20	-	0.32	0.56	396.5	465.0	2.070
W-80	+	0.63	0.54	117.1	0.0	2.124
W-99	-	0.54	0.22	2199.4	1211.2	2.298
W-33	+	0.67	0.55	1279.6	1279.6	2.380
W-2	-	0.89	0.59	956.0	1152.3	2.462
W-50	-	0.74	0.18	2349.0	1016.1	2.507
W-38	-	1.00	0.53	1605.7	1434.4	2.547
W-107	+	0.10	0.60	126.7	126.7	2.563
W-97	-	0.81	0.71	639.9	651.2	3.281
W-123	-	1.00	0.00	1904.0	1877.1	3.290
W-148	-	0.05	0.34	1130.3	2172.4	3.349
W-162	+	0.40	0.70	307.4	800.5	3.370
W-14	-	1.00	0.72	1245.8	1229.3	3.535
W-132	-	1.00	0.63	1746.9	1781.9	3.591
W-176	+	0.67	0.67	1702.2	1702.2	3.713
W-59	-	0.68	0.49	329.6	1982.2	3.888
W-7	-	0.83	0.71	1761.3	1642.0	3.952
W-173	-	0.08	0.75	163.7	168.4	3.995
W-6	-	1.00	0.72	1947.2	1722.0	4.235
W-131	-	1.00	0.29	2785.6	146.5	4.324
W-34	-	0.55	0.64	1685.3	2159.1	4.363

W-157	-	1.00	0.80	734.4	165.4	4.400
W-44	-	1.00	0.83	555.7	566.7	4.564
W-158	-	1.00	0.80	1327.6	167.9	4.604
W-108	-	0.83	0.78	1816.7	1961.6	4.947
W-156	-	0.95	0.88	1268.9	1289.9	4.996
W-45	-	1.00	0.85	1722.3	1760.0	5.207
W-137	+	0.32	0.90	881.9	1292.2	5.382
W-30	-	1.00	0.92	1118.5	1339.8	5.510
W-40	-	1.00	0.92	997.4	287.4	5.686
W-15	-	1.00	0.93	563.6	556.4	5.761
W-82	-	0.83	0.62	2513.6	2739.4	6.157
W-58	-	1.00	1.00	1061.2	1074.5	6.397
W-62	-	1.00	1.00	979.5	966.7	6.405
W-61	-	0.74	1.00	968.4	1006.0	6.413
W-41	-	1.00	1.00	1682.1	1155.3	6.453
W-136	-	1.00	1.00	903.9	622.7	6.499
W-49	-	1.00	1.00	1469.3	618.2	6.588
W-31	-	1.00	1.00	2126.9	1323.4	6.744
W-172	-	0.84	0.91	2349.1	2349.1	6.988
W-72	-	0.57	1.00	76.8	678.8	7.076
W-53	+	1.00	1.00	70.1	408.6	7.126
W-142	+	0.91	1.00	1518.9	199.3	7.154
W-64	+	1.00	1.00	181.4	197.8	7.154
W-56	-	0.89	1.00	352.9	1356.5	7.158
W-37	-	1.00	1.00	1313.5	1967.9	7.172
W-48	-	0.87	0.92	2251.5	2477.5	7.289
W-77	-	0.84	1.00	2454.4	2168.5	7.619
W-8	-	1.00	0.75	3770.5	2282.7	7.948
W-57	-	0.93	0.76	3988.7	1792.4	8.318
W-79	-	0.81	1.00	2316.9	2694.5	8.568

W-17	-	1.00	1.00	1768.8	2682.1	8.573
W-65	+	1.00	0.58	3278.5	3325.1	8.767
W-159	+	0.48	0.00	3249.1	3319.5	10.052
W-169	-	0.92	0.31	4381.6	411.7	10.056
W-88	+	0.81	0.40	3621.1	3543.5	10.083
W-11	-	1.00	1.00	4247.6	2094.5	10.893
W-125	-	1.00	0.00	3413.1	3468.7	11.013
W-67	+	0.69	0.71	4375.4	210.7	11.892
W-74	-	1.00	1.00	3912.4	263.2	12.380
W-171	-	0.92	0.90	4087.5	117.8	12.482
W-28	-	1.00	1.00	1532.6	3716.4	12.715
W-75	-	1.00	1.00	251.9	3453.0	13.980
W-39	+	1.00	0.70	4461.4	4090.6	14.097
W-78	-	0.65	0.84	2153.9	4433.2	14.695
W-68	-	0.93	1.00	567.4	3981.0	16.052
W-10	-	0.89	0.85	4208.7	4857.0	17.708
W-149	-	1.00	1.00	6341.3	999.0	22.093
W-76	-	1.00	1.00	208.9	4787.3	22.633
W-167	-	0.48	0.00	3102.7	5630.5	24.594
W-116	+	0.91	0.00	5281.6	5373.2	26.408
W-81	-	0.79	1.00	11081.8	1371.6	62.924

Ref. A postulated reference with the best overall resistance.

Table S4. Technical Note: The Conditions of Mass Spectrometry Parameters.

Name	Parent	Product	SRM Collision Energy	Retention Time	Time Window	Polarity
DON	297.1	91.1	40	5	8	+
DON	297.1	175.2	15	5	8	+
3-ADON	339	63.1	19	5	8	+

3-ADON	339	266.3	23	5	8	+
15-ADON	339	63	16	5	8	+
15-ADON	339	137.1	5	5	8	+
D3G	503.1	427.2	22	5	8	-
D3G	503.1	457.3	18	5	8	-

Limit of determination is 1 μg/kg.; +, positive ion mode; -, Negative ion mode.
